# Surface and Functional Properties of Biosurfactant Produced by *Bacillus rugosus* HH2 Derived from *Jeotgal*

**DOI:** 10.4014/jmb.2505.05029

**Published:** 2025-07-18

**Authors:** Geum-Jae Jeong, Do-Kyun Kim, Kyung-Jin Cho, Eun-Jin Choi, Won-Kyo Jung, Fazlurrahman Khan, Young-Mog Kim

**Affiliations:** 1 Department of Food Science and Technology, Pukyong National University, Busan 48513, Republic of Korea; 2 Marine Integrated Biomedical Technology Center, The National Key Research Institutes in Universities, Pukyong National University, Busan 48513, Republic of Korea; 3 Research Center for Marine Integrated Bionics Technology, Pukyong National University, Busan 48513, Republic of Korea; 4 Major of Biomedical Engineering, Division of Smart Healthcare, College of Information Technology and Convergence and New-senior Healthcare Innovation Center (BK21 Plus), Pukyong National University, Busan 48513, Republic of Korea

**Keywords:** *Bacillus rugosus*, biosurfactant, surface properties, functional properties

## Abstract

Surfactants play a critical role in diverse industrial applications. However, concerns over the environmental persistence and toxicity of synthetic variants have prompted a shift toward sustainable biosurfactants. In this study, the surface properties of a biosurfactant produced by *Bacillus rugosus* HH2 isolated from *Jeotgal*, a traditional Korean fermented seafood, were investigated to evaluate its industrial applicability. The biosurfactant exhibited consistent and stable emulsifying performance across a wide range of salinity (3–18%), temperature (30–80°C), and pH (4–10) conditions. Emulsification assays with various hydrocarbon substrates revealed a performance equal to or better than that of the synthetic surfactant Triton X-100. While the biosurfactant generated a lower initial foam volume than Triton X-100, it maintained a significantly higher foam stability over 60 min, highlighting its suitability for applications that benefit from persistent and low-volume foaming. Optical microscopy showed that the biosurfactant produced smaller and more uniform bubbles than those of Triton X-100. Furthermore, the producing strain demonstrated strong hydrophobic interactions with hydrocarbons, such as hexadecane, toluene, and pyrene, supporting its potential utility in hydrocarbon-rich environments. Collectively, these findings highlight the multifunctional surface and functional activity and environmental robustness of the *B. rugosus* HH2-derived biosurfactant, indicating its potential as a sustainable and effective alternative for diverse industrial applications, including food processing, cosmetics, and environmental remediation.

## Introduction

Surfactants are characterized by their amphiphilic nature, comprising a hydrophilic (polar) head and a hydrophobic (nonpolar) tail, which allows them to reduce surface and interfacial tensions between immiscible phases such as liquid–liquid or liquid–solid systems [1]. Because of these physicochemical properties, surfactants are extensively utilized in diverse industrial sectors, including the food processing, agricultural, cosmetic, pharmaceutical, and petrochemical sectors [2]. Traditionally, most surfactants have been synthesized from petrochemical feedstock through industrial chemical processes [3]. However, growing concerns regarding the environmental persistence, poor biodegradability, and potential toxicity of synthetic surfactants have prompted increased interest in sustainable alternatives [4]. To address these challenges, biosurfactants have attracted considerable attention as environmentally benign substitutes. These surface-active compounds are biosynthesized by diverse microbial species, such as bacteria, yeasts, and fungi—and are typically used as secondary metabolites during microbial growth on renewable substrates [5]. Structurally, biosurfactants are amphiphilic and comprise a hydrophobic fatty acid moiety and hydrophilic head group, which may include carbohydrates, amino acids, or phosphate residues [3]. Compared to their synthetic counterparts, biosurfactants exhibit several advantages, such as high biodegradability, low toxicity, and functional stability under extreme conditions, including high salinity, temperature fluctuations, and pH variability [5, 6]. Moreover, biosurfactants typically exhibit lower critical micelle concentrations (CMC) compared to synthetic surfactants, which enhances their efficiency across various applications [7]. These attributes highlight the potential of biosurfactants not only as effective surface-active agents, but also as key components in the development of green technologies aligned with principles of the circular bioeconomy [8].

The structural and functional characteristics of biosurfactants are often influenced by the microbial strains and their environmental origins [9]. Among various producers, species of the genus *Bacillus* have attracted considerable attention because of their capacity to synthesize a wide array of potent biosurfactants, including surfactin, bacillomycin, fengycin, and lichenysin [10]. *Bacillus* strains are frequently recovered from industrial or polluted environments (*e.g.*, soil, sludge, and wastewater), which facilitate efficient biosurfactant production and present potential biosafety risks, thereby limiting their suitability for use in pharmaceutical or food-related applications [11]. Conversely, *Bacillus* strains isolated from fermented food products are generally recognized as safe and are often associated with probiotic properties, including enhanced biocompatibility and superior bioactivity [12, 13]. These food-derived strains demonstrate promising potential for producing biosurfactants that not only maintain surface-active capabilities but also meet the stringent safety standards required for biomedical and nutraceutical applications [11, 12].

In a previous study, a biosurfactant-producing strain, *B. rugosus* HH2 was isolated from *Jeotgal*, a traditional Korean fermented seafood [13]. Structural characterization revealed that the biosurfactant produced by *B. rugosus* HH2 was primarily composed of surfactin homologs. The biosurfactant effectively reduced the surface tension of water to 37.63 ± 1.15 mN/m at a CMC of 0.1 mg/ml and exhibited a concentration-dependent decrease in cell surface hydrophobicity. Thermal stability assays demonstrated that the biosurfactant remained stable at temperatures up to 145°C, and the emulsion formed from the culture supernatant retained high durability even under extreme environmental conditions. Notably, the biosurfactant exhibited strong antibacterial, antibiofilm, and antivirulence activities against *Staphylococcus aureus* without significant *in vitro* cytotoxicity or *in vivo* phytotoxicity at effective concentrations. These findings suggest that the biosurfactant derived from *B. rugosus* HH2 holds promise as a multifunctional agent for pharmaceutical, cosmetic, and food industry applications, particularly as an antimicrobial or emulsifying compound.

Accordingly, the isolation of *Bacillus* species from fermented foods for the recovery of biosurfactants is a promising strategy for a wide range of industrial applications. Previous studies have focused primarily on the antibacterial effects of biosurfactants produced by *B. rugosus* HH2 against *S. aureus* [13]. However, to advance its industrial applicability, an in-depth understanding of its surface and functional properties, including stability, emulsifying capacity, foaming ability, and hydrophobicity, is essential because these factors significantly influence its functionality at application-relevant concentrations [7]. Therefore, this study aimed to comprehensively assess the surface and functional activity of a biosurfactant derived from *B. rugosus* HH2 isolated from *Jeotgal*. In addition, its functional potential was evaluated through a comparative analysis with synthetic surfactants.

## Materials and Methods

### Chemicals

Benzene, chloroform, hexadecane, hydrochloric acid, methanol, paraffin, petroleum ether, pyrene, sodium chloride, sodium hydroxide, toluene, Triton X-100, and xylene were purchased from Sigma-Aldrich (USA). Aviation turbine fuel (ATF), engine oil, olive oil, and soybean oil were purchased from the local market. Tryptic soy agar (TSA) and tryptic soy broth (TSB) were purchased from Difico (USA). Phosphate-buffered saline (PBS) was purchased from Gibco (USA). The solvents used in all the procedures were of certified analytical grade.

### Bacteria Strain and Biosurfactant Production

The biosurfactant-producing strain *B. rugosus* HH2 was isolated from *Jeotgal* and identified using 16S rRNA gene sequencing [13]. The sequence was deposited in GenBank under the accession number PQ157569. For preservation, the strain was cultured on TSA at 37°C and subcultured biweekly to maintain viability.

To produce the biosurfactant, the procedure outlined by Zargar *et al*. [14] was adapted with minor modifications. *B. rugosus* HH2 was cultured in TSB at 37°C with agitation (150 rpm) for five days to promote biosurfactant synthesis. Upon completion of incubation, the culture was centrifuged at 12,000 rpm for 20 min at 4°C to remove the microbial biomass. The clarified supernatant was then passed through a 0.2 μm pore-size membrane to yield a cell-free extract. Biosurfactant precipitation was initiated by lowering the pH of the filtered supernatant to 2.0 through the dropwise addition of 6 M HCl. The acidified mixture was left to rest overnight at 4°C, allowing amphiphilic molecules to aggregate and settle. The following day, the resulting precipitate was collected by centrifugation under identical conditions and reconstituted in a minimal volume of deionized water. The suspension was then neutralized to pH 7.0 using 1 M NaOH for solvent extraction. The biphasic solvent extraction was performed by slowly introducing a 2:1 (v/v) mixture of chloroform and methanol. This process was continued until the final volumetric ratio of chloroform:methanol:water reached 8:4:3. After agitation overnight, the upper organic phase containing the biosurfactant was isolated, and the solvents were removed under reduced pressure using a rotary evaporator. The remaining fraction was freeze-dried to obtain crude biosurfactant powder, which was stored at –20°C for further experimentation.

### Biosurfactant Stability

The stability of the biosurfactant under a broad range of salinity, temperature, and pH conditions was evaluated with slight modifications to the method described by Ahmad *et al*. [15]. The biosurfactant solution was prepared in sterile distilled water at a CMC of 0.1 mg/ml [13]. To assess salinity tolerance, 3 ml of the biosurfactant solution was supplemented with sodium chloride (NaCl) at varying concentrations (3.0, 6.0, 9.0, 12.0, 15.0, and 18.0%, w/v). Subsequently, mixing with 3 ml of engine oil for 1 min was conducted to form emulsions. These emulsions were incubated at 25°C for 24 h [16]; then, the emulsification index (EI) was recorded. For temperature stability, emulsions were prepared by mixing 3 ml of the biosurfactant solution with 3 ml of engine oil for 1 min. Samples were incubated at different temperatures (30, 40, 50, 60, 70, and 80°C) for 24 h, followed by the recording of EI measurements. To determine pH-dependent stability, the pH of the 3 ml biosurfactant solution was adjusted to 4.0, 5.0, 6.0, 8.0, 9.0, and 10.0 using 1.0 mol/l HCl or NaOH. Each pH-adjusted sample was mixed with 3 ml of engine oil for 1 min to generate emulsions. After incubation at 25°C for 24 h, the EI was measured. The EI was calculated as follows: EI (%) = [height of the emulsion layer (cm)/total height of the liquid column (cm)] × 100.

### Emulsifying Capacity

The emulsifying activity of the biosurfactant toward various hydrocarbon substrates was evaluated with slight modifications to the method described by Rehman *et al*. [17]. A 3 ml aliquot of the biosurfactant solution prepared at its CMC was mixed with 3 ml of different immiscible hydrocarbons—including ATF, benzene, olive oil, paraffin, petroleum ether, soybean oil, toluene, and xylene—via vortexing for 1 min. The EI was determined by measuring the height of the emulsion layer within 2 min (day 0) and after incubation at room temperature for 24 h (day 1) [16]. Triton X-100 at its CMC (0.22 mM) was used as a control.

### Foam Generation Capacity

The foam-generation ability of the biosurfactant was evaluated by slightly modifying the procedure reported by Huang *et al*. [18]. A 10 ml portion of the biosurfactant solution, prepared at its CMC, was placed into a sealed cylindrical vial and subjected to vigorous agitation using a vortex mixer for 2 min. The foam height was recorded at specific intervals, within 2 min (initial) and after 5, 30, and 60 min, to monitor foam formation and stability. The foam structure induced by the biosurfactant was visualized using an optical microscope (CX23RTFS2; Olympus, Japan). Foaming ability was calculated as follows: Foaming ability (%) = [foam layer height (cm)/total column height (cm)] × 100. For comparison, a CMC-level solution of Triton X-100 was used as a control.

### Bacterial Adhesion to Hydrocarbons

The cell surface hydrophobicity of the biosurfactant-producing strain was assessed using the bacterial adhesion to hydrocarbons method, as described by Das [19]. *B. rugosus* HH2 was cultivated in TSB for 24 h, followed by centrifugation at 12,000 rpm for 15 min. The harvested bacterial cells were washed twice with PBS and then resuspended in PBS to an optical density (OD) of 0.5–0.6 at 600 nm. A 5 ml aliquot of the bacterial suspension was mixed with 500 μl of various hydrocarbons (hexadecane, toluene, and pyrene) and allowed to stand undisturbed for 30 min to facilitate phase separation. Subsequently, the OD of the aqueous phase was measured at 600 nm. Cell surface hydrophobicity was calculated using the following equation: Cell surface hydrophobicity (%) = [(OD_0_ –OD_1_) / OD_0_] × 100, where OD_0_ is absorbance of the bacterial suspension before hydrocarbon addition, and OD1 is absorbance of the aqueous phase after the hydrocarbon treatment.

### Statistical Analysis

All experiments were conducted in triplicate. Data were analyzed using a one-way analysis of variance, followed by Dunnett’s post-hoc test using GraphPad Prism version 10.4.2. Results are presented as means ± standard deviation. Statistical significance was defined as *p<0.05. “ns” denotes no significant difference.

## Results and Discussion

### Influence of Salinity, Temperature, and pH on Biosurfactant Stability

**Fig. 1:**
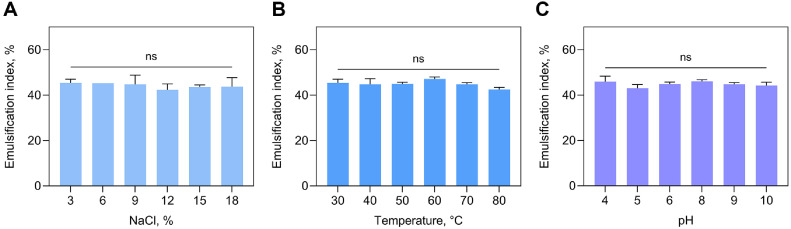
Evaluation of biosurfactant stability in response to (A) salinity, (B) temperature, and (C) pH variations. ns means no significance.

To ensure the broad industrial applicability of biosurfactants, it is essential to thoroughly evaluate their functional stability under a wide range of salinity, temperature, and pH conditions. In this study, emulsification stability was evaluated using a biosurfactant solution at its CMC under these stress conditions. To determine the effect of salinity, EI values were measured after adding different concentrations of NaCl to the biosurfactant solutions prepared at the CMC (Fig. 1A). Even at a high NaCl concentration of 18%, the EI values remained within a stable range (42.33–45.35%), with no statistically significant differences observed (*p* > 0.05). This salinity tolerance suggests that the biosurfactant may be suitable for applications in high-salinity food-processing conditions and marine bioremediation settings [20, 21]. The thermal stability of the biosurfactant was further examined by evaluating its emulsification performance at temperatures ranging from 30–80°C (Fig. 1B). Across this temperature range, the EI values remained consistently high (42.52–47.12%) without significant variations (*p* > 0.05), indicating excellent heat resistance. Such thermal robustness is advantageous for applications involving heat treatment, including high-temperature biotechnological processes, such as microbial-enhanced oil recovery [22]. To assess pH tolerance, the biosurfactant’s emulsification activity was tested across a wide pH range (4.0–10.0) (Fig. 1C). The EI values showed minimal fluctuations (43.02–45.98%) throughout the tested range, indicating consistent performance under both acidic and alkaline conditions (*p* > 0.05). This pH stability supports the biosurfactant’s use as an emulsifier or stabilizer in the food and cosmetics industries, where exposure to variable pH conditions is common [7, 23]. Collectively, these results demonstrate that the biosurfactant derived from *B. rugosus* HH2 maintained its emulsification efficiency across a wide spectrum of salinity, temperature, and pH conditions, highlighting its potential for practical use in diverse industrial and environmental applications.

### Emulsifying Capacity with a Range of Hydrocarbon Substrates

**Fig. 2:**
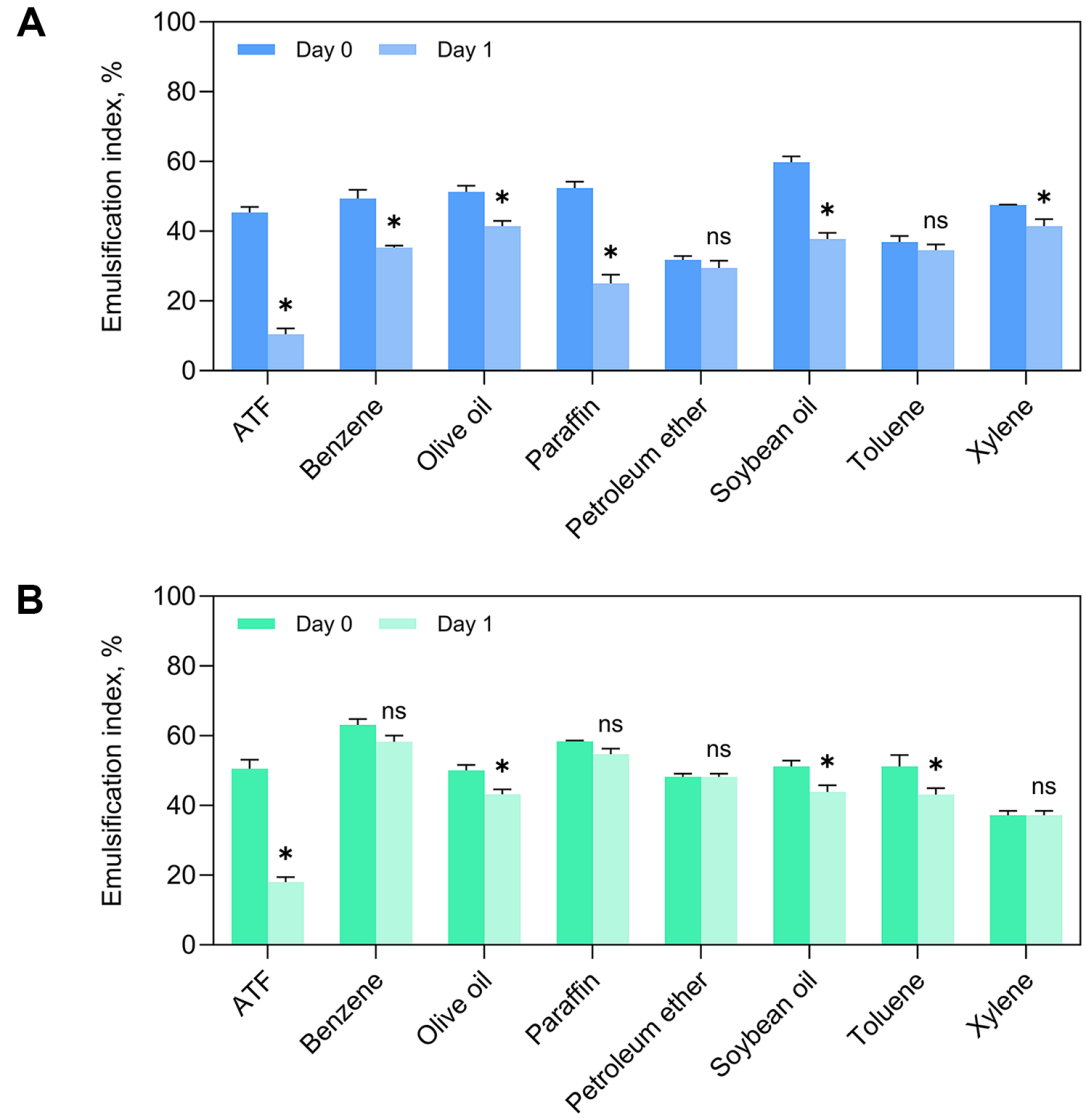
Assessment of emulsifying ability across diverse hydrocarbon substrates. (**A**) Biosurfactants and (**B**) Triton X-100. Asterisk (*) indicates a statistically significant difference at the *p* < 0.05. ns means no significance.

The ability of biosurfactants to form emulsions with a variety of hydrocarbon substrates is a key feature of their potential applications in diverse industrial sectors [5]. The emulsifying activity of the biosurfactant produced by *B. rugosus* HH2 was assessed using several hydrocarbons and oils (Fig. 2A). At its CMC, the biosurfactant rapidly formed emulsions (within 2 min) with ATF, benzene, olive oil, paraffin, petroleum ether, soybean oil, toluene, and xylene, achieving EI values ranging from 31.76% to 59.76%. Among these, the highest EI was observed with soybean oil (59.76 ± 1.72%), indicating a superior emulsifying capacity. The broad emulsifying spectrum of the biosurfactant was comparable to that of the synthetic surfactant Triton X-100 (Fig. 2B), suggesting its potential for wide-ranging industrial applications [7]. However, Triton X-100 exhibited higher EI values than the biosurfactant for most substrates, except olive oil, soybean oil, and xylene, with EI values ranging between 37.23% and 63.10%. This pattern is consistent with previous reports showing that synthetic surfactants typically displayed higher emulsification efficiencies than those of biosurfactants [24]. Notably, the biosurfactant exhibited strong and stable emulsification with plant-derived oils, highlighting its suitability as a natural emulsifier for food-related applications [23]. In addition, no significant difference (*p* > 0.05) was observed between the initial and 24 h EI values for emulsions formed with petroleum ether and toluene, suggesting excellent short-term performance due to the absence of rapid emulsion breakdown with these short-chain hydrocarbons. This finding underscores the potential of the *B. rugosus* HH2-derived biosurfactant for use in petroleum-related bioremediation processes [25]. However, the decrease in EI after 24 h may be influenced not only by the stability of the biosurfactant itself, but also by physical instability of the emulsion or subtle environmental changes that could affect micelle integrity [26, 27]. These aspects should be taken into account when considering potential industrial applications. In conclusion, the biosurfactant produced by *B. rugosus* HH2 displayed emulsifying capabilities comparable to those of the synthetic surfactant Triton X-100, supporting its potential as a sustainable alternative for various industrial and environmental applications.

### Foam Generation Behavior of the Biosurfactant

**Fig. 3:**
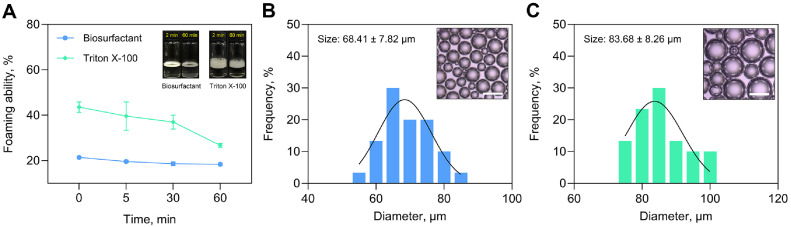
Evaluation of the biosurfactant’s foaming behavior. (**A**) Foaming ability of biosurfactants and Triton X-100. Optical micrographs and corresponding bubble size distributions produced by (**B**) biosurfactants and (**C**) Triton X-100. The white scale bar represents 100 μm.

Surfactants facilitate foam formation by adsorbing at the air–liquid interface, where they lower surface tension, thereby reducing the energy barrier for bubble creation [28]. The foaming performance of the biosurfactant produced by *B. rugosus* HH2 was evaluated and compared with that of the synthetic surfactant, Triton X-100 (Fig. 3A). The biosurfactant achieved a foaming rate of 21.40 ± 0.95% within 2 min, which was a timeframe notably lower than that of Triton X-100 (43.55 ± 2.28%). Although a high foaming capacity is advantageous in certain contexts, surfactants with lower foam generation capacities are often more appropriate for such applications as bioremediation, industrial cleaning, and solvent extraction [29]. The temporal stability of the foam produced using the biosurfactant was assessed at multiple time points. After 5, 30, and 60 min, the foaming rates were 19.60 ± 0.57%, 18.60 ± 0.85%, and 18.40 ± 0.57%, respectively, resulting in a foam retention rate of 85.98%. By contrast, Triton X-100 exhibited a progressive decline to 39.60 ± 6.22%, 36.96 ± 3.08%, and 26.71 ± 0.89% at the corresponding time points, equating to a stability of 61.33%. Foam morphology was examined using optical microscopy. The biosurfactant generated relatively uniform, spherical bubbles with an average diameter of 68.41 ± 7.82 μm (Fig. 3B), while Triton X-100 produced larger and more heterogeneous bubbles averaging 83.68 ± 8.26 μm (Fig. 3C). This finding suggests a higher degree of polydispersity in the foam structure of the synthetic surfactant, which may negatively affect foam longevity and quality. The superior foam stability of the biosurfactant is likely attributable to its molecular characteristics. Previous studies linked effective foam stabilization to amphiphilic molecules with hydrophobic alkyl chains [18]. These features are consistent with surfactin, the principal lipopeptide found in the biosurfactant produced by *B. rugosus* HH2 [13], which demonstrated excellent foam retention over time and formed more uniform bubbles than Triton X-100 did. These attributes highlight its potential utility in industries that require stable foaming conditions with a minimal bulk foam volume.

### Cell Surface Hydrophobicity of the Biosurfactant-Producing Bacteria

Cell surface hydrophobicity plays a crucial role in determining bacterial adhesion to water–hydrocarbon interfaces, thereby influencing interactions with hydrophobic organic compounds [17]. The hydrophobicity of *B. rugosus* HH2 toward various hydrocarbons is summarized in Table 1. The strain exhibited adhesion levels of 53.58 ± 1.21% to hexadecane, 60.39 ± 0.77% to toluene, and 52.68 ± 0.45% to pyrene, demonstrating a strong affinity for diverse hydrocarbon substrates [29]. These values were comparable to those reported for *Pseudomonas furukawaii* PPS-19, which showed adhesion rates ranging from 51.27% to 60.63% for crude oil, n-dodecane, and pyrene [19]. Enhanced hydrophobic interactions in biosurfactant-producing bacteria are often attributed to structural changes in the cell envelope, mediated by biosurfactant adsorption [30]. This process masks hydrophilic functional groups on the cell surface, thereby increasing overall surface hydrophobicity [17]. Collectively, these findings suggest that *B. rugosus* HH2 has a strong affinity for hydrocarbons, supporting its potential as a promising biosurfactant-producing strain.

## Conclusion

This study investigated the surface and functional characteristics of a biosurfactant produced by *B. rugosus* HH2, a strain isolated from *Jeotgal*, to assess its potential industrial applications. The biosurfactant maintained consistent emulsification performance across a broad range of environmental factors, including high salinity (3–18%), temperature variations (30–80°C), and pH levels (4–10), indicating strong environmental tolerance. It showed effective emulsification with a variety of hydrocarbons, particularly plant-based oils and short-chain hydrocarbons, demonstrating an emulsifying efficiency comparable to that of the synthetic surfactant Triton X-100. These characteristics suggest its versatility in sectors that require eco-friendly emulsifying agents. Foaming tests showed that, although the biosurfactant generated less initial foam than that of Triton X-100, it exhibited significantly greater stability over time, which is beneficial for applications requiring persistent and low-volume foaming. Furthermore, cell surface hydrophobicity assays showed that *B. rugosus* HH2 exhibited a high degree of affinity for hydrocarbons, such as hexadecane, toluene, and pyrene, reflecting its inherent capability for hydrocarbon interaction and biosurfactant production. Collectively, these findings indicate that the biosurfactant produced by *B. rugosus* HH2 combines functional effectiveness with environmental stability, supporting its potential as a naturally derived sustainable substitute for conventional synthetic surfactants in a range of industrial applications.

## Figures and Tables

**Table 1:** Assessment of cell surface hydrophobicity of *Bacillus rugosus* HH2 against various hydrocarbons.

Hydrocarbons	Cell surface hydrophobicity (%)
Hexadecane	53.58 ± 1.21
Toluene	60.39 ± 0.77
Pyrene	52.68 ± 0.45

## References

[1] Bolan S, Padhye LP, Mulligan CN, Alonso ER, Saint-Fort R, Jasemizad T (2023). Surfactant-enhanced mobilization of persistent organic pollutants: potential for soil and sediment remediation and unintended consequences. J. Hazard. Mater..

[2] Thakur V, Baghmare P, Verma A, Verma JS, Geed SR (2024). Recent progress in microbial biosurfactants production strategies: applications, technological bottlenecks, and future outlook. Bioresour. Technol..

[3] Muñoz SS, Balbino TR, Alba EM, Barbosa FG, de Pier FT, de Almeida ALM (2022). Surfactants in biorefineries: role, challenges & perspectives. Bioresour. Technol..

[4] Manga EB, Celik PA, Cabuk A, Banat IM (2021). Biosurfactants: opportunities for the development of a sustainable future. Curr. Opin. Colloid Interface Sci..

[5] Kumar PS, Ngueagni PT (2021). A review on new aspects of lipopeptide biosurfactant: types, production, properties and its application in the bioremediation process. J. Hazard. Mater..

[6] Sharma J, Sundar D, Srivastava P. 2023. *Advantages and disadvantages of biosurfactants over other synthetic surfactants*, pp. 505-523. Ed. Springer.

[7] Jahan R, Bodratti AM, Tsianou M, Alexandridis P (2020). Biosurfactants, natural alternatives to synthetic surfactants: physicochemical properties and applications. Adv. Colloid Interface Sci..

[8] Mgbechidinma CL, Akan OD, Zhang C, Huang M, Linus N, Zhu H (2022). Integration of green economy concepts for sustainable biosurfactant production-a review. Bioresour. Technol..

[9] Shekhar S, Sundaramanickam A, Balasubramanian T (2015). Biosurfactant producing microbes and their potential applications: a review. Crit. Rev. Environ. Sci. Technol..

[10] Gaur VK, Sharma P, Gupta S, Varjani S, Srivastava J, Wong JW (2022). Opportunities and challenges in omics approaches for biosurfactant production and feasibility of site remediation: strategies and advancements. Environ. Technol. Innov..

[11] Hajfarajollah H, Eslami P, Mokhtarani B, Akbari Noghabi K (2018). Biosurfactants from probiotic bacteria: a review. Biotechnol. Appl. Biochem..

[12] Gayathiri E, Prakash P, Pratheep T, Ramasubburayan R, Thirumalaivasan N, Gaur A (2024). Bio surfactants from lactic acid bacteria: an in-depth analysis of therapeutic properties and food formulation. Crit. Rev. Food Sci. Nutr..

[13] Jeong GJ, Kim DK, Park DJ, Cho KJ, Kim MU, Oh DK (2024). Control of *Staphylococcus aureus* infection by biosurfactant derived from *Bacillus rugosus* HH2: strain isolation, structural characterization, and mechanistic insights. J. Hazard. Mater..

[14] Zargar AN, Lymperatou A, Skiadas I, Kumar M, Srivastava P (2022). Structural and functional characterization of a novel biosurfactant from *Bacillus* sp. IITD106. J. Hazard. Mater..

[15] Ahmad Z, Zhang X, Imran M, Zhong H, Andleeb S, Zulekha R (2021). Production, functional stability, and effect of rhamnolipid biosurfactant from *Klebsiella* sp. on phenanthrene degradation in various medium systems. Ecotoxicol. Environ. Saf..

[16] Satpute SK, Banpurkar AG, Dhakephalkar PK, Banat IM, Chopade BA (2010). Methods for investigating biosurfactants and bioemulsifiers: a review. Crit. Rev. Biotechnol..

[17] Rehman R, Ali MI, Ali N, Badshah M, Iqbal M, Jamal A (2021). Crude oil biodegradation potential of biosurfactant-producing *Pseudomonas aeruginosa* and *Meyerozyma* sp. J. Hazard. Mater..

[18] Huang H, Li Z, Ma Y, Yao M, Yao S, Zhang Z (2023). High-performance arabinoglucuronoxylan-based biosurfactants for oily sludge separation. Carbohydr. Polym..

[19] Das S (2023). Cell surface hydrophobicity and petroleum hydrocarbon degradation by biofilm-forming marine bacterium *Pseudomonas furukawaii* PPS-19 under different physicochemical stressors. J. Hazard. Mater..

[20] Aparna A, Srinikethan G, Smitha H (2012). Production and characterization of biosurfactant produced by a novel *Pseudomonas* sp. 2B. Colloids Surf. B Biointerfaces.

[21] Al-Sakkaf MK, Onaizi SA (2023). Crude oil/water nanoemulsions stabilized by rhamnolipid biosurfactant: effects of acidity/basicity and salinity on emulsion characteristics, stability, and demulsification. Fuel.

[22] Zhang J, Gao H, Xue Q (2020). Potential applications of microbial enhanced oil recovery to heavy oil. Crit. Rev. Biotechnol..

[23] Jui AH, Hossain MN, Afrin S, Bhowmik B, Ranadheera CS, Bhuiyan MHR. 2025. Microbial biosurfactants: prospect and challenges for application in food industry. *Food Rev. Int.* 1-34. https://doi.org/10.1080/87559129.2025.2478199. 10.1080/87559129.2025.2478199

[24] Pornsunthorntawee O, Wongpanit P, Chavadej S, Abe M, Rujiravanit R (2008). Structural and physicochemical characterization of crude biosurfactant produced by *Pseudomonas aeruginosa* SP4 isolated from petroleum-contaminated soil. Bioresour. Technol..

[25] Mishra S, Lin Z, Pang S, Zhang Y, Bhatt P, Chen S (2021). Biosurfactant is a powerful tool for the bioremediation of heavy metals from contaminated soils. J. Hazard. Mater..

[26] Langevin D (2023). Recent advances on emulsion and foam stability. Langmuir.

[27] Owen SC, Chan DP, Shoichet MS (2012). Polymeric micelle stability. Nano Today.

[28] Wan ZL, Wang LY, Wang JM, Yuan Y, Yang XQ (2014). Synergistic foaming and surface properties of a weakly interacting mixture of soy glycinin and biosurfactant stevioside. J. Agric. Food Chem..

[29] Pandey R, Krishnamurthy B, Singh HP, Batish DR (2022). Evaluation of a glycolipopepetide biosurfactant from *Aeromonas hydrophila* RP1 for bioremediation and enhanced oil recovery. J. Clean. Prod..

[30] Krasowska A, Sigler K (2014). How microorganisms use hydrophobicity and what does this mean for human needs?. Front. Cell. Infect. Microbiol..

